# Inverse association of natural mentoring relationship with distress mental health in children orphaned by AIDS

**DOI:** 10.1186/1471-244X-10-6

**Published:** 2010-01-16

**Authors:** Francis N Onuoha, Tsunetsugu Munakata

**Affiliations:** 1 Department of Human Care Science, Graduate School of Comprehensive Human Sciences University of Tsukuba, Tsukuba, Japan

## Abstract

**Background:**

The magnitude of the AIDS-orphaned children crisis in sub-Saharan Africa has so overstretched the resource of most families that the collapse of fostering in the sub-region seems imminent (UNICEF, 2003), fueling the need for a complementary/alternative care. This paper examines the probability of the natural mentoring care to ameliorate distress mental health in children orphaned by AIDS.

**Methods:**

952 children, mean age about 14 years, from local community schools and child-care centers in Kampala (Uganda) and Mafikeng/Klerksdorp (South Africa) towns participated in the study. The design has AIDS-orphaned group (n = 373) and two control groups: Other-causes orphaned (n = 287) and non-orphaned (n = 290) children. We use measures of child abuse, depression, social discrimination, anxiety, parental/foster care, self-esteem, and social support to estimate mental health. Natural mentoring care is measured with the Ragins and McFarlin (1990) Mentor Role Instrument as adapted.

**Results:**

AIDS-orphaned children having a natural mentor showed significant decreased distress mental health factors. Similar evidence was not observed in the control groups. Also being in a natural mentoring relationship inversely related to distress mental health factors in the AIDS-orphaned group, in particular. AIDS-orphaned children who scored high mentoring relationship showed significant lowest distress mental health factors that did those who scored moderate and low mentoring relationship.

**Conclusions:**

Natural mentoring care seems more beneficial to ameliorate distress mental health in AIDS-orphaned children (many of whom are double-orphans, having no biological parents) than in children in the control groups.

## Background

Orphan children tend to manifest more depression [1], personality disorder [2], and anxiety/insomnia [3] tendencies than do non-orphans. These orphan children may present psychosomatic symptoms [3] and health worries [4] that may impede positive mental health. Material and emotional supports from parents during childhood may have enduring psychosocial health benefits [5]. These parental supports, which the orphan child may lack, fulfill the affective function of the family to its members [6,7]. Orphans may encounter hopelessness, and frustration [8] often owing to their new circumstance that may require them to not only fend for themselves but also for their younger ones, in some cases. However, Abebe and Aase [9] tend to disagree. They argue that the symptomatic perception of orphans rests on stereotyping: most orphans have shown the resilience to get on with the challenges of life following parental death [9]. Other authors [10] report higher generalized anxiety disorder from children living in parents' separated homes than from orphans.

Chitiyo, Changara, and Chitiyo [11] suggest that children orphaned by AIDS may be unique orphans. They tend to grief long before parental death(s) owing to the debilitating AIDS-defining illnesses that may precede death. Due to the moral shame associated with HIV infection [11], AIDS-orphaned children may encounter higher stigma/social discrimination than do other orphan categories [12]. According to UNAIDS, UNICEF, and USAID [13]:

*"An especially important and distinctive characteristic of HIV/AIDS in regard to orphaning is that AIDS is more likely than other causes of death to create double orphans. With HIV/AIDS, if one parent is infected there is a higher probability that the other parent is or will become infected and that both will eventually die .... Surveys consistently show that double orphans are more disadvantaged than single orphans" *(p.11).

Subbarao, Mattimore, and Plangemann [14] identify several care options for the mental health need of the African orphan child. Prominent among them is the "normative" fostering practice [15], in which parents may allow their children to be reared elsewhere for kinship or economic gains. For children orphaned by AIDS, "crisis" fostering [16] is the typical, in which moral obligations may compel one to take-in children having no parents. Foster children, however, tend to be unfairly treated in food allocation, domestic chores allocation, and school attendance that may adversely affect mental health [17]. What is more, in contemporary times, the magnitude of the AIDS-orphaned crisis seems to so overstretch the resources of families in sub-Saharan Africa that the collapse of fostering seems imminent [18], necessitating the need for a support/alternative care system.

The purpose of the present study is to estimate the association of being in a natural mentoring relationship care with mental health in AIDS-orphaned children. Natural mentorship is different from organizational mentoring [19] which is common in the workplace. Natural mentoring is provided in homes and communities [20] by adult figures [21], such as the local school teacher, local elders, the church pastor, neighbors, etc, and extended family members who may exert influences on children as surrogate-parents [22].

Natural mentoring care is different from fostering care, in which the child tends to emigrate from her biological home to the fosterer's. In natural mentorship such dislocation is not required. The dyad relationship may not be conflict-free, but a range of its psychosocial benefits such as risk behavior control [23], personality adjustment [24], and social resilience [25] has been reported, suggesting its usefulness for orphan population.

## Methods

### Procedure

There was a pilot preceding the present study to validate the study instruments in the African countries. In keeping with the *UN Convention on the Rights of the Child: its relevance for social scientists *[26], the study protocols satisfied the ethical requirements of confidentiality, anonymity, and voluntary participation [27].

We visited nine community schools, and six NGO child support centers at Mafikeng/Klerksdorp areas (North-West Province, South Africa) and Kampala district (Uganda) to conduct the survey. The UN definition of orphanhood as the loss of one or both parents [13] is adopted; so is the UN definition of a child as persons aged below 18 used. Local interviewers are Luganda (Uganda) and Setswana/Afrikaans (South Africa) speaking research collaborators. The interviewer-administered questionnaire method is adopted for low education children; otherwise, the self-report method was dominantly used. The interview duration lasts approximately 45 minutes per session at the end of which the child receives a ball pen.

### Participants

The study participants are 952 children (Uganda = 459; South Africa = 492) in 3 groups: AIDS-, other-causes, and non-orphaned children. *AIDS-orphaned group*: We ask: Is your father/mother living (Yes/No)? If not living, what is the cause of death? Response choices: "1. HIV/AIDS, 2. War, 3. Others, 4. Don't know." Children who check HIV/AIDS are assigned to this group (n = 373). Owing to the shame associated with HIV infection, children may feign ignorance of HIV-related cause of parent's death [11,28]. We assign to this group children who answered "don't Know" to the cause of parent's death, if both parents are deceased, in consonance with the UN *essential *characteristics of AIDS orphaning [13]. A negligible few children are also assigned to the group utilizing the "verbal autopsy" [29] accounts of the community school/child support center heads, as explained elsewhere. *Other-causes orphaned group: *Children who check "war/others" are assigned to this group (n = 289). Those whose parents are living form the *non-orphan group *(n = 290).

### Measures

#### Mental health

Mental health is estimated by the combination of anxiety, depression, social discrimination, child abuse, self-esteem, social support, and parental/foster care scales.

The Anxiety subscale of the renowned General Health Questionnaire (GHQ-28 (30)) is utilized to measure *anxiety*. The 6-item subscale (alpha = .81) negatively (r = -.34, p < .01) correlates with Rosenberg's Self Esteem, and positively (r = .40 p < .01, respectively) with the CES-DC (Center for Epidemiological Studies Depression Scale for Children) [31] as adapted. The response categories on the scale are scored from 0 (never) to 3 (always) in which expected maximum score is 18.

*Depression *is estimated with the CES-DC [31]. The test-retest reliability and concurrent validity for the CES-DC are adequate [32]. We utilize the first 10 items (somatic complaints, 5 items; negative affects, 3 items; and positive affects, 2 items) of the 20-item CES-DC. To strengthen the internal stability of the measure to alpha = .77, we exclude the two positive affect items during analysis. Sample items on the 8-item CES-DC include: "I was bothered by things that usually don't bother me; I didn't feel like eating, I wasn't very hungry; I wasn't able to feel happy, even when friends tried to make me feel good; I felt like I was too tired to do things." Response scores range from 0 (never) to 3 (always).

##### Perceived social support

We adapt 6-items (alpha = .83) from the 15-item Schwarzer and Schulz [33] Received Support Scale to estimate social support. The measure positively associates with Rosenberg's Self-esteem (r = .36, p < .01) and negatively with the GHQ-28 Anxiety (r = -.38, p < .01). The measure requires the respondent to "think about person(s) that is closest to you - your friend(s), guardian(s) or parent(s)/foster parent(s) - how does this person treat you?" For example: S/he "is there when I need him/her; shows love to me; takes care of my financial needs; in general, I am satisfied with the way s/he treats me." Responses are scored from 0 (never) to 3 (always).

##### Self-esteem

The Rosenberg Self-Esteem [34] Scale that measures favorable or unfavorable regard for the self is the most utilized measure of self-esteem [35]. The Cronbach alpha for the Scale in the present study is .60, which compares favorably with the value found by Lorenzo-Hernandez and Ouellette [36]. The RSE shows discriminant validity against anxiety (r = -.34, p < .01), and social discrimination (r = -.40, p < .01).

##### Social discrimination

We utilize the modified Detroit Area Study Measure of Discrimination [37] to estimate social discrimination (alpha = .78). Typical questions are: In your daily life, *compared to other people around you, do you: *Feel differently treated? Feel unfairly treated? Made to feel inferior? Prevented from doing things others are allowed to do? People behave as though they are afraid of you? The measure appreciably correlate with depression (r = .38, p < .01), social support (r = -.25, p < .01) and child abuse (r = .30, p < .01).

##### Child Abuse

We ask 4 questions, each of which estimates the physical, verbal, sexual, and labor dimensions of child abuse [38]: Are you - physically beaten in a manner you consider unfair; verbally abused in a manner you consider unfair; forced to "sleep"/have sex with anyone; forced against your wish to work on the farm for someone? Responses are scored from 3 (always) to 0 (never). The alpha reliability of the measure, which discriminate depression (r = .21, p < .01) and perceived social support (r = -.36, p < .01) is .76.

*Parental/foster care *is measured with the Parental Bonding Instrument (PBI) [39]. The 25-item PBI assesses both parental care and over-protection. The "care" dimension estimates empathy, affection, warmth, and independence. "Over-protection" comprises parental intrusion, infantilism, and control. Support for the reliability and validity of the PBI as a measure of actual and perceived parenting has been reported [40]. We utilize 8 items in the "care" subscale (alpha = .86) in the present study. Typical items include parents/foster parents: are affectionate to me; understand my problems and worries; let me do things I enjoy doing; enjoy discussing things with me; give me as much freedom as I want. Responses are scored from 0 (never) to 3 (always), higher scores representing higher care.

*Distress mental health factors *(alpha = .87) is the summation of child abuse, depression, social discrimination, and anxiety scores. *Positive mental health factors *(alpha = .86) is the sum of parental/foster care, perceived social support, and self-esteem scores.

#### Natural mentoring relationship

In consonance with the operational definition of natural mentoring [21,25], we ask the participants: Other than your parent(s) or foster parent(s) is there any adult person(s) in the neighborhood you go to for support and guidance for most things you do (Yes/No)? If "Yes," how often do you meet this person (0 = rarely, 1 = sometimes, 2 = often, 3 = very often)? Children who answer "Yes", and check any of *1--3 *meeting frequencies are classified as being in a mentoring relationship. These children (n = 714) rate the Ragins' Mentor Role Instrument (MRI) that estimates parental, modeling, counseling, friendship, and support roles by mentors to mentees. Children not in a mentoring relationship form the control group. The 33-item MRI [41] measure has 11 mentor roles of 3 items each on a 7-point likert response of 1 (strongly disagree) to 7 (strongly agree). We exclude the 6 workplace-related *formal *mentor roles (ie, job sponsorship, coaching, protection, challenge, exposure and socialization), and utilize the 5 *informal *roles (ie, parenting, counseling, modeling, acceptance, and friendship) each of which is estimated with 2 items on a 4-point likert response score of 0 (never) to 3 (always). The internal stability of the adapted MRI is alpha = .91, which is similar to the value found by Ragins and Cotton [42]. The instrument, which shows discriminant validity against anxiety (r = -.158, p < .01) and social support (r = .379, p < .01), has the following sample items: Treats me as a son/daughter (parental role); represents who I want to be (modeling role); guides me to choose the career I want (counseling role); provides me support and encouragement (friendship); acts as a leader to me (acceptance). Expected score range is 0-30, higher scores suggest higher impact of mentorship on the child.

### Analysis

**Figure 1:**
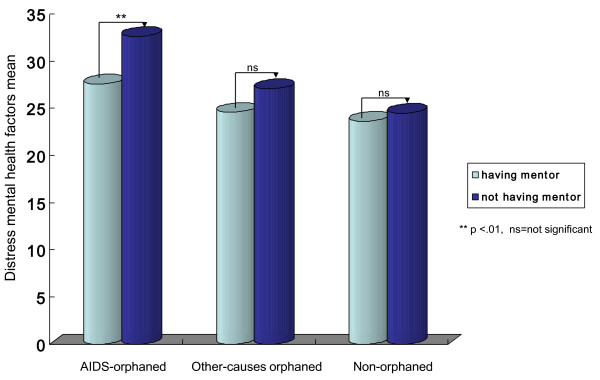
**ANOVA of having/not-having a natural mentor for each of the 3 groups showing significant higher distress mental health factors in AIDS-orphaned children without natural mentors**.

The Pearson's measure of association shows admissible discriminant validity of the study measures. The Chronbach alpha shows sufficient reliability. We separate children who report being in mentoring relationship (n = 714) from those who do not (n = 234) to perform the ANOVA of distress mental health between them in each of the 3 groups (Figure 1). To examine the association of mentoring relationship with distress mental health factors, we ranked scores of the perceived impact of mentoring relationship as *low (scores 0-10), moderate (11-20)*, and *high (21-30) *and examined their performance on mental health in the 3 groups (Table 1). To estimate performance by orphan-types (ie no parents versus single-parents), we performed the ANOVA of having and not having a natural mentor in the two orphan types (Table 2).

**Table 1:** ANOVA showing significant inverse asociation of mentoring relationship with distress mental health in the AIDS-orphaned group

		**AIDS-orphaned**			**Other-causes orphaned**			**Non-orphaned**		
				
**Factors**	**MR**	**n**	**M(SD)**	**Posthoc**	**n**	**M(SD)**	**Posthoc**	**n**	**M(SD)**	**Posthoc**
			
Child abuse	1	113	3.57(2.95)		98	3.28(3.10)		13	2.23(2.77)	
	2	101	2.91(2.88)		68	2.87(3.05)		54	2.13(2.41)	
	3	157	2.32(2.47)	1>2^d^,1>3*,2>3^d^	117	2.14(2.57)	1>2^d^,1>3*,2>3^d^	141	1.67(2.42)	1>2^d^,1>3^d^,2>3^d^
										
Depression	1	113	11.33(5.64)		98	9.59(5.15)		13	7.38(4.15)	
	2	102	10.42(4.21)		69	9.67(4.60)		54	8.48(4.13)	
	3	158	9.93(4.71)	1>2^d^,1>3^d^,2>3^d^	118	9.80(5.01)	1<2^d^,1<3*,2<3^d^	141	9.76(5.74)	1<2^d^,1<3^d^,2<3^d^
										
Social discrimination	1	113	7.01(4.63)		98	5.51(3.79)		13	4.62(4.81)	
	2	101	6.10(3.40)		68	5.57(3.77)		54	4.98(3.35)	
	3	157	5.64(3.65)	1>2^d^,1>3*,2>3^d^	118	4.83(3.85)	1<2^d^,1>3^d^,2>3^d^	141	6.43(4.84)	1<2^d^,1<3^d^,2<3^d^
										
Anxiety	1	113	9.15(5.28)		98	6.55(4.50)		13	4.85(3.05)	
	2	101	7.70(4.56)		68	6.69(4.11)		54	6.04(3.25)	
	3	157	6.80(4.31)	1>2^d^,1>3*,2>3^d^	117	5.50(3.77)	1<2^d^,1>3^d^,2>3^d^	141	5.14(3.79)	1<2^d^,1<3^d^,2>3^d^
										
Parental/foster care	1	113	8.37(5.53)		98	11.20(6.66)		13	11.77(5.67)	
	2	101	10.79(5.27)		68	12.00(4.91)		54	11.87(4.51)	
	3	157	13.75(6.03)	1<2,1<3*,2<3*	118	14.10(5.63)	1<2^d^,1<3*,2<3^d^	141	15.60(5.36)	1<2^d^,1<3^d^,1<3*
										
Self-esteem	1	113	13.93(4.75)		98	15.90(5.18)		13	17.38(4.81)	
	2	102	14.96(4.88)		69	15.50(4.14)		54	16.94(4.56)	
	3	158	16.51(4.58)	1<2^d^,1<3*,2<3*	118	17.10(4.57)	1<2^d^,1<3^d^,2<3^d^	141	17.98(4.41)	1>2^d^,1<3^d^,2<3^d^
										
Social support	1	113	6.08(4.08)		98	8.12(4.53)		13	8.77(3.75)	
	2	101	7.60(3.89)		66	8.98(3.23)		53	8.49(4.01)	
	3	157	9.92(4.24)	1<2*,1<3*,2<3*	118	11.00(3.76)	1<2^d^,1<3*,2<3*	141	11.45(3.29)	1>2^d^,1<3^d^,2<3*
										
Distress mental health	1	113	32.73(13.40)		98	26.40(12.58)		13	21.31(8.77)	
	2	102	28.51(10.20)		69	25.90(11.66)		54	23.13(8.58)	
	3	158	25.96(10.80)	1>2*,1>3*,2>3*	118	23.70(11.21)	1>2^d^,1>3^d^,2>3^d^	141	24.23(11.80)	1<2^d^,1<3^d^,2<3^d^
										
Positive mental health	1	113	32.73(13.40)		98	26.40(12.58)		13	21.31(8.77)	
	2	102	33.16(11.50)		69	35.80(10.17)		54	37.31(10.30)	
	3	158	39.77(11.70)	1<2*,1<3*,2<3*	118	42.10(10.26)	1<2^d^,1<3^d^,2<3*	141	44.78(8.83)	1>2^d^,1<3^d^,2<3*

^d ^not significant, * p < .05, MR = mentoring relationship scores: 1 = low, 2 = moderate, 3 = high

**Table 2:** ANOVA showing differences of having and not-having a natural mentor on mental health by orphan-types

	Orphan-types									
	No parents					Single-parents				
						
	Having natural mentor		Not having natural mentor			Having natural mentor		Not having natural mentor		
				
Variables	n	M (SD)	n	M (SD)	*F*	n	M (SD)	n	M (SD)	*F*
Child abuse	258	2.76 (2.75)	61	3.57 (3.15)	4.12*	242	2.45(2.65)	100	3.62(3.18)	12.16**
Depression	260	9.99 (4.80)	61	10.59 (6.01)	0.7^d^	244	10.00(4.77)	100	10.86(4.95)	2.23^d^
Social discrimination	258	5.86 (3.55)	61	6.67 (5.04)	2.19^d^	243	5.35(3.93)	100	6.38(3.89)	4.86*
Anxiety	258	7.17 (4.37)	61	8.70 (5.78)	5.29*	242	6.40(4.30)	100	7.66(4.70)	5.75*
Parental/foster care	258	11.87 (6.19)	61	9.67 (6.17)	6.22*	243	12.60(5.59)	100	10.66(6.51)	7.77**
Self-esteem	260	15.50 (4.55)	61	13.89 (4.75)	6.12*	244	16.48(4.78)	100	15.35(5.01)	3.87*
Social support	258	8.53 (4.39)	61	6.85 (4.35)	7.23**	241	9.66(4.06)	100	7.51(4.44)	18.82**
Distress mental health factors	260	27.07 (10.92)	61	31.15 (14.91)	5.93*	244	25.60(11.52)	100	30.12(12.64)	10.30*
Positive mental health factors	260	35.72 (12.27)	61	30.33 (11.45)	9.77**	244	38.38(11.47)	100	33.38(12.33)	12.88**

^d ^not significant, *p < .05 **p < .01

## Results

### Demographic outcome

373 AIDS-orphaned, 285 other-causes orphaned and 290 non-orphaned children validly participate in the study. The majority (94%) of the children are aged 10 to 17 years. Grand mean age is 13.59 years (SD = 2.34). There are no significant difference (F = .259(2), p = .77) of age in the groups: Mean = 13.54 (SD = 2.52), 13.55 (SD = 2.11), and 13.67 (SD = 2.32) for AIDS-, other-causes, and non-orphaned children, respectively. No significant educational level variance (F = 1.96(2), p = .14) in the 3 groups is observed. There is no gender influence on mental health outcomes. Male and female children scored similar levels of distress/positive mental health outcomes in the study and control groups.

### Mental health outcomes

AIDS-orphaned children in a natural mentoring relationship show significant lower distress mental health factors (child abuse, social discrimination, anxiety, and depression) than did their counterparts not in a mentoring relationship. Similar significant association are unobserved in the control groups (Figure 1). Also natural mentoring relationship show inverse relationship to distress mental health: AIDS-orphaned children who score *low *mentoring relationship show significant high distress mental health factors than do *moderate *and *high *mentoring AIDS-orphaned children (Table 1). In the control groups, variances in the relationship are not significant. The association of having a mentor or not with mental health do not vary by orphan types (Table 2). In both orphan types, single-parent and no-parent orphans having a natural mentor have lower distress mental health factors, suggesting the psychosocial usefulness of mentoring to both AIDS- and other-causes induced orphaning.

## Discussion

Wickrama, Lorenz, and Conger [43] report that children who receive parental social support (caring, acceptance, and assistance) show fewer psychosomatic symptoms. For AIDS-orphaned children, who are more likely than other-causes orphaned children to encounter double parental loss (or double loss of parental support), the consequence of orphaning may be graver. Children orphaned by AIDS, in the present study, show significant higher anxiety, lower self-esteem, lower social support, and lower positive mental health factors than do those in the control groups. Reasons for the situation may be ascribed to double orphaning [13,44-47]. Double orphans in this study, whether by AIDS- or other-causes show similar levels of psychological health. Their levels of high child abuse, depression, social discrimination, anxiety, and low foster parental care, self-esteem, social support seem statistically the same, suggesting that they share common psychosocial circumstance. Double-orphaned children in the present study show significant lower self-esteem, social support, and positive mental health factors than do single-orphaned.

In most domains of the distress mental health construct, having a natural mentor show significant inverse association with distress mental health factors in the AIDS-orphaned group. Children orphaned by AIDS who score *high *impact of mentoring relationship score significant *lower *distress mental health factors than do AIDS-orphans who score *moderate *and *low *mentorship. In the control groups, the variances are weak, suggesting that natural mentoring relationship may be stronger to ameliorate distress mental health factors in AIDS-orphaned children, many of whom have no parents.

Natural mentoring relationship seems more psychosocially beneficial to orphans than to non-orphans. For example, whereas an inverse association of mentoring and distress health is seen in the two orphan groups, the reverse seems the case for non-orphaned children. In this group, high mentoring shows tendencies to elicit high distress mental health factors (Table 1). The reason for the outcome is not clear, although parental censorship of children's mentoring relationship may be likely. In orphans, whether double- or single-orphaned, having a natural mentor show beneficial effects to reduce distress and increase positive mental health factors in them.

Age shows inverse relationship to natural mentorship in all the groups. Younger children significantly engage in higher mentoring relationship than do older children. These younger children significantly regarded their mentors as a father, mother or role model than do older children.

## Conclusions

Ideally, natural mentors should be biologically unrelated, nonparent others. But in the traditional African social environment, a thin line may exist between natural mentors and extended family kins. Most of the natural mentors in the present study are extended family kins rather than non-family members. Natural mentorship does not require the mentee to live with the mentor as the case in fostering. The scenario may mean greater independence for the protégé and lesser social burden for the mentor. Natural mentors have been used to strengthen psychosocial well-being in child-headed households, who are victims of intra-state genocide [48]. In children orphaned by AIDS, natural mentoring relationship seems beneficial to reduce distress mental health factors.

## Study limitations

Our study design is cross-sectional. Perhaps an anthropological design that participatorily investigates the mentoring behaviors of the mentee and mentor *over a time *may produce a more meaningful result.

We are unable to absolutely vouch for the AIDS-orphaned category. Death certificates are unreliable medical data [12] in most AIDS-stigmatizing African countries. Cluver et al. [12] review the "verbal autopsy" method validated in several sub-Saharan African studies [29] to determine cause of parental death. The method require the presence of observable AIDS-defining illnesses such as oral candidiasis, Kaposi's sarcoma and the HIV wasting syndrome [49]. However, the distinctive characteristic of HIV/AIDS in regard to orphaning is that AIDS is more likely than other causes of death to create double orphans [13]. We combined the UN double orphan criterion, the children's self-report, and verbal autopsy reports from the local school/child support center heads to construct the AIDS-orphaned group.

The natural mentors in the study are not interviewed. We posit that the omission may not adversely affect the study outcome. If the child rates the social milieu between her and her natural mentor as positive, it seems likely that the natural mentor may so positively perceive the social environment.

## Competing interests

The authors declare that they have no competing interests.

## Authors' contributions

FNO and TM jointly conceptualized and concretized the study. Both authors read and approved the final manuscript.

## Pre-publication history

The pre-publication history for this paper can be accessed here:

http://www.biomedcentral.com/1471-244X/10/6/prepub
